# Synapse-specific roles for microglia in development: New horizons in the prefrontal cortex

**DOI:** 10.3389/fnmol.2022.965756

**Published:** 2022-08-08

**Authors:** Sara V. Blagburn-Blanco, Megan S. Chappell, Lindsay M. De Biase, Laura A. DeNardo

**Affiliations:** ^1^Neuroscience Interdepartmental Program, University of California, Los Angeles, Los Angeles, CA, United States; ^2^Medical Scientist Training Program, University of California, Los Angeles, Los Angeles, CA, United States; ^3^Department of Physiology, University of California, Los Angeles, Los Angeles, CA, United States

**Keywords:** microglia, mPFC, synapse, development, neurodevelopmental disorder (NDD)

## Abstract

Dysfunction of both microglia and circuitry in the medial prefrontal cortex (mPFC) have been implicated in numerous neuropsychiatric disorders, but how microglia affect mPFC development in health and disease is not well understood. mPFC circuits undergo a prolonged maturation after birth that is driven by molecular programs and activity-dependent processes. Though this extended development is crucial to acquire mature cognitive abilities, it likely renders mPFC circuitry more susceptible to disruption by genetic and environmental insults that increase the risk of developing mental health disorders. Recent work suggests that microglia directly influence mPFC circuit maturation, though the biological factors underlying this observation remain unclear. In this review, we discuss these recent findings along with new studies on the cellular mechanisms by which microglia shape sensory circuits during postnatal development. We focus on the molecular pathways through which glial cells and immune signals regulate synaptogenesis and activity-dependent synaptic refinement. We further highlight how disruptions in these pathways are implicated in the pathogenesis of neurodevelopmental and psychiatric disorders associated with mPFC dysfunction, including schizophrenia and autism spectrum disorder (ASD). Using these disorders as a framework, we discuss microglial mechanisms that could link environmental risk factors including infections and stress with ongoing genetic programs to aberrantly shape mPFC circuitry.

## Introduction

The medial prefrontal cortex (mPFC) controls complex cognitive and emotive functions, including decision making, memory, social interactions and mood (Euston et al., 2012). These functions mature throughout a prolonged developmental window, lasting until around postnatal day (P) 60 in mice and the mid-twenties in humans. During this period, a combination of genetic programs and activity-dependent processes refine mPFC circuits (Schubert et al., 2015; Klune et al., 2021). Developmental challenges occurring on a background of genetic risk factors can alter mPFC synapse and circuit maturation. For instance, infection or stress increase the risk of developing neurodevelopmental disorders such as autism spectrum disorder (ASD) (Estes and McAllister, 2015) and schizophrenia (SZ) (Müller et al., 2015).

Microglia are increasingly recognized as key players in synapse development and refinement (Thion et al., 2018; Dorman and Molofsky, 2019). Microglial progenitors enter the brain and spinal cord during embryogenesis, and, through migration and proliferation, ubiquitously colonize the CNS parenchyma (De Biase et al., 2017; Bachiller et al., 2018). Microglia survey their local environment by extending motile processes that interact with nearby cells and sample local signaling molecules (Bachiller et al., 2018; Marinelli et al., 2019). In turn, microglia shape normal circuit maturation (Thion et al., 2018; Zengeler and Lukens, 2021) by regulating aspects of axon guidance (Squarzoni et al., 2014), axon myelination, synapse formation (Gallo et al., 2022), synapse maturation (Hoshiko et al., 2012), and synapse refinement (Schafer et al., 2012; Schafer and Stevens, 2015; Stevens and Schafer, 2018). Because they are exquisitely sensitive to changes in neural activity, inflammation, and stress, microglial support for circuit development can be perturbed by numerous CNS insults (Hanamsagar and Bilbo, 2017). Indeed, microglia exhibit major transcriptional, morphological, and functional changes in many neuropsychiatric disorders including ASD and SZ (Rahimian et al., 2021), suggesting that altered microglial-neuron interactions may critically link genetic risk and early insults to long-term changes in circuit function. More detailed knowledge of how microglia shape mPFC circuit maturation may enable us to effectively intervene when the developmental balance is shifted toward neuropsychiatric disorders.

Microglia express a wide array of membrane-bound receptors that equip them to sense damage-associated molecules, pathogens, chemokines, complement system proteins, purines, and neurotransmitters (Noda et al., 2000; Hickman et al., 2013; Krauthausen et al., 2014; Marinelli et al., 2019). Stimulation of these receptors causes functional changes in microglia by altering their motility, physiological properties (Kreutzberg, 1996; Gomez-Nicola and Perry, 2015), phagocytic activity, and/or cytokine production (Noda et al., 2000; Kuhn et al., 2004). Microglial secreted factors can then act on neuronal and astrocyte receptors to influence processes such as dendritic spine formation and elimination (Parkhurst et al., 2013), insertion of α-amino-3-hydroxy-5-methyl-4-isoxazolepropionic acid (AMPA) receptors at synapses (Heir and Stellwagen, 2020), and membrane excitability (Marinelli et al., 2019; Badimon et al., 2020). Foundational studies also demonstrated that microglial phagocytic function can target synaptic material, supporting physiological elimination of excess synapses during development (Paolicelli et al., 2011; Schafer et al., 2012). Microglia recognize complement system proteins deposited on synapses that signal “eat me” or “don’t eat me” *via* membrane-bound receptors. This represents one key mechanism by which microglia engulf synaptic compartments (Schafer et al., 2012; Lehrman et al., 2018; Borucki et al., 2020). Collectively, these receptor-ligand interactions exemplify the complex multi-directional communication between cell populations that characterizes CNS development.

Cellular heterogeneity adds a layer of complexity layer of complexity to the mechanisms that support physiological and perturbed circuit maturation. While regional and cell-to-cell variation in neuronal function is widely accepted, the heterogeneity of microglia has only recently been appreciated. Microglia exhibit regional differences in morphology, density, gene expression, cell process motility, and phagocytic activity (De Biase et al., 2017; Ayata et al., 2018; Bachiller et al., 2018; Stowell et al., 2019; Hope et al., 2020), suggesting that their impact on synapse and circuit function likely varies. Moreover, the maturation of microglial populations varies across brain regions (Squarzoni et al., 2014), as do the mechanisms they use to execute synapse pruning (Gallo et al., 2022). This underscores the need to carefully define microglial roles in circuit development on a circuit-by-circuit basis.

Understanding the roles of microglia in mPFC circuit development is of particular importance because of the region’s strong ties to neuropsychiatric disorders. A recent study in rats showed that mPFC spine density peaks around P39, a time that aligns with maximal microglial engulfment of postsynaptic material (Mallya et al., 2019). A second recent study showed that depletion of mPFC microglia in mid-adolescence (P42–P47)—but not in adulthood—reduced dendritic complexity, excitatory synaptic density, and mushroom spine density and induced impairments in cognitive behavioral functions in adults (Schalbetter et al., 2022). Adolescent depletion also increased inhibitory synaptic transmission in adults. Although these studies did not examine molecular mechanisms underlying observed microglial-neuron interactions, their findings indicate that mid-adolescence is a sensitive period when microglia can influence cognitive development by refining mPFC circuits, and that microglia regulate distinct mPFC circuit elements depending on the developmental stage.

In the following sections of this review, we discuss novel findings elucidating how microglia and immune signals regulate synaptogenesis and activity-dependent synaptic refinement, primarily in sensory circuits. We then consider how the glial pathways that sculpt sensory cortices might shape mPFC connectivity during its prolonged development. Finally, we discuss the role of microglia in disease and highlight how disruptions to glia-neuron interactions are implicated in the pathogenesis of neurodevelopmental and psychiatric disorders associated with mPFC dysfunction, including schizophrenia and ASD.

## New players in complement-dependent regulation of microglial function

Complement cascade proteins, which are classically used to “tag” pathogens for phagocytic removal by immune cells, can regulate the elimination of supernumerary synapses during development (Stevens et al., 2007; Schafer et al., 2012; Sekar et al., 2016). Key examples of complement-mediated synapse tagging include the “eat me” signal C1q, which is deposited at presynaptic terminals of less-active synapses. C1q then binds and activates C3, forming a complex that can be recognized by the microglial complement receptor 3 (CR3), and provokes synapse engulfment. Although the C1q-C3-CR3 pathway has been shown to regulate synapse removal in multiple contexts, including the developing lateral geniculate nucleus (Schafer et al., 2012), neurodegenerative disease (Hong et al., 2016), and infection (Vasek et al., 2016), many gaps remain in our understanding of the role that complement plays in microglial-synapse interactions. How is the system balanced by “don’t eat me” signals? CD47, which is recognized by microglial receptor Sirp1α, can inhibit microglial synaptic phagocytosis (Lehrman et al., 2018), but are there others? How exactly does C1q become attached to synapses displaying appropriate activity patterns, as microglia themselves appear to be the main producers of C1q (Fonseca et al., 2017; Vukojicic et al., 2019; Burger et al., 2020)? Finally, microglial-based synapse pruning happens independently of the C1q-C3-CR3 pathway in some brain regions (Gunner et al., 2019; Kovács et al., 2021), raising questions about how many distinct mechanisms govern microglial synapse pruning across different brain regions.

**FIGURE 1:**
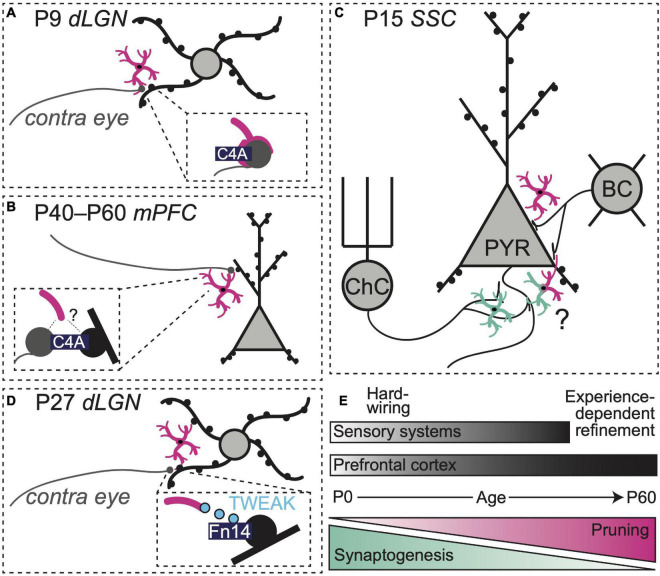
Region-, synapse-, and age-specific roles for microglia in synapse refinement. **(A)** Microglia prune retinogeniculate synapses at P9 using a C4-dependent pathway (Yilmaz et al., 2021). **(B)** Overexpression of C4 enhances synapse pruning in mPFC (Yilmaz et al., 2021). **(C)** At P15, microglia prune axosomatic synapses between basket cells and pyramidal cells (Favuzzi et al., 2021) while promoting the formation of axo-axonic synapses between chandelier cells and pyramidal cells (Gallo et al., 2022) in rodent somatosensory cortex. **(D)** Neuronal Fn14 together with microglial TWEAK refine dLGN dendritic spines through activity-dependent, non-phagocytic mechanisms (Cheadle et al., 2020). **(E)** Synaptogenesis and refinement overlap in space and time and occur over distinct windows of development in sensory systems vs. prefrontal cortex.

Within the complement system, additional regulatory players may also be involved. The complement protein C4 localizes to synapses (Presumey et al., 2017), but its role in synaptic pruning has proven challenging to define. Intriguingly, genetic variants of C4, including alleles that increase C4 copy number, are strongly associated with an increased risk of SZ (Sekar et al., 2016). Loss of cortical gray matter and dendritic spine abnormalities have also been noted in SZ patients, raising the possibility that aberrant synapse pruning by microglia during adolescence is an underlying pathological mechanism driving disease progression (Sekar et al., 2016). A novel transgenic mouse expressing variable copy numbers of human *C4A* (*hC4A*) and *C4B* recently shed light on the molecule’s role in synaptic refinement within the retinogeniculate system (Yilmaz et al., 2021). Within this new humanized mouse model, C4A overexpression (8 copies) boosted microglial synapse engulfment and reduced synaptic density in the dorsal lateral geniculate nucleus (dLGN) of the thalamus during the postnatal window when contralateral and ipsilateral eye inputs to this region are undergoing refinement (P9–10, Figure 1A).

The same study also observed synapse density changes in mPFC, a region implicated in the pathogenesis of SZ in humans (Lewis, 1997; Volk and Lewis, 2013; Yilmaz et al., 2021). However, these effects were observed later compared to the visual system. Elevated hC4A copy number (8 alleles) increased C4 protein above control levels at P10 and to a lesser extent at P40. The increase in C4 protein in mPFC preceded an elevation in microglial phagocytic activity and pre-synaptic material engulfment, which peaked during adolescence (P40). A reduction in cortical synapse density was not apparent until early adulthood (P60, Figure 1B), indicating that effects of C4 deposition on microglial activity are cumulative and not instantaneous. Adult mice overexpressing *hC4A* exhibited deficits in social interactions, increased anxiety-like behaviors, and decreased performance in tasks relying on spatial working memory. Notably, these behaviors have been previously linked to mPFC circuits (Adhikari et al., 2011; Gee et al., 2016; Ko, 2017; Gangopadhyay et al., 2021) and are reminiscent of negative symptoms of schizophrenia in humans (Correll and Schooler, 2020). Interestingly, a loss of C4 protein did not alter spine density in the mPFC, nor did it induce behavioral deficits, suggesting that alternative or redundant pathways contribute to normal maturation of synapses within the frontal cortex.

Recent work using patient-derived microglial-like cells (iMGs) provided new insight into mechanisms through which C4-dependent pathways sculpt synapses (Sellgren et al., 2019). In this study, iMGs effectively engulfed synaptosomes and their presence reduced spine density in co-cultured human-derived neurons. Strikingly, iMGs from schizophrenia patients showed higher levels of phagocytic activity than those derived from a healthy control population. This difference in engulfment was largely explained by features inherent to patient iMGs, and partially modulated by synaptic factors. There was a positive correlation between *C4AL* alleles, C3 protein deposition on synapses, and levels of synaptic engulfment. This suggests that *C4AL*, a known risk allele for SZ, may induce pathological circuit alterations in patients by promoting excessive amounts of C3-dependent microglial phagocytosis.

Collectively, these studies illustrate how genetic variation that shifts C4 abundance could be connected to disease *via* aberrant microglial pruning. It is possible that yet undiscovered genetic variation in other pruning-relevant molecules (either *via* altered copy number or alterations in enhancers) also contributes to disease risk or etiology. As such, we need a more precise understanding of how and when these molecules shape microglial pruning. It will be important to determine whether biological events can elevate C4 expression during adolescence or provoke C4 production outside of its developmentally appropriate time window, leading to pathological circuit modifications *via* microglial effectors. Individuals with a higher *C4A* copy number may exhibit particularly strong increases in C4 in certain situations, and, therefore, experience increased vulnerability in particular contexts. These findings also raise the intriguing possibility that an individual’s risk of developing schizophrenia might be mitigated *via* interventions that modify microglial pruning activity. Along this vein, minocycline, an antibiotic previously shown to reduce synapse pruning in rodent models (Nutile-McMenemy et al., 2007; Schafer et al., 2012; Inta et al., 2017), elicited a dose-dependent reduction of synaptosome engulfment *in vitro*. Moreover, a small-scale analysis of electronic health records suggested that adolescents who received chronic doses of minocycline exhibited a reduced risk of incident psychosis (Sellgren et al., 2019). Though these small-scale analyses of patient data are promising, future prospective studies and randomized trials will be necessary to assess the drug’s therapeutic potential. Stratifying or analyzing clinical study cohorts by risk variants with a known impact on microglial biology may also be crucial for uncovering a candidate treatment’s true efficacy.

## Microglial regulation of inhibitory synapse development

Synapses are heterogeneous. Distinct expression of synthetic enzymes, membrane-bound receptors, and cell-adhesion proteins at pre and postsynaptic terminals helps specify connectivity between neurons and determines their function within a larger circuit as either excitatory or inhibitory units (Shen and Scheiffele, 2010; Yogev and Shen, 2014). Most work examining synaptic interactions of microglia has focused on excitatory synapses (Paolicelli et al., 2011; Schafer et al., 2012). However, recent work revealed a synaptogenic role for microglia at inhibitory synapses [defined by their expression of ionotropic or metabotropic gamma-aminobutyric acid (GABA) receptors] in postnatal somatosensory cortex in mice (Gallo et al., 2022). The authors showed that microglia promote the formation and maintenance of specialized axo-axonic synapses between inhibitory chandelier cells and the axon-initial segments (AIS) of pyramidal neurons (PyNs) in the somatosensory cortex. Microglial depletion from P7–P18 reduced the number of inhibitory boutons at these specialized chandelier synapses. Importantly, removing GABA B1 receptors (GABAB1Rs) from microglia lowered the frequency of microglia-PyN contacts and diminished the density of inhibitory AIS-associated synapses on these neurons. These observations demonstrate that microglial GABAB1Rs influence—through yet unknown pathways—the synaptogenic interactions between AIS associated-microglia, chandelier cells, and PyNs.

A second recent study identified a key role for microglia in inhibitory synapse pruning during early postnatal development, also in the mouse somatosensory cortex (Favuzzi et al., 2021). While a global depletion of microglia from P1–15 increased the density of both excitatory and inhibitory synapses, deleting GABAB1Rs from microglia only impacted inhibitory synapses—namely, axo-somatic synapses between parvalbumin-positive (PV +) basket cells and PyNs. Together with spatial transcriptomics analyses, these observations suggest that approximately 30% of microglia in the developing somatosensory cortex express GABA1Rs and activation of these receptors regulates microglial interactions with inhibitory synapses. GABAB1R deletion from microglia altered expression of numerous genes, including those associated with synapse pruning and phagocytosis. These observations raise questions about whether GABAB1R expression allows microglia to locate and engage with inhibitory synapses directly or whether activation of these receptors—possibly from tonic local GABA tone—shapes the overall phenotype of the cell, including its phagocytic profile, *via* regulation of gene expression.

Taken together, these studies indicate that microglia shape the development of inhibitory synapses and highlight the importance of microglial GABAB1Rs in modulating microglial interactions with this specific subset of synapses (Figure 1C). Future studies should seek to elucidate the signaling pathways downstream of microglial GABA receptor activation to clarify how these receptors can promote phagocytosis of synaptic targets in one developmental context and exert synaptogenic effects in another. It also remains to be seen if and when similar GABAR-dependent mechanisms are at play in mPFC development. In general, the question of whether neurotransmitter receptor expression by microglia confers specificity to microglia-synapse interactions also bears further investigation. Do individual GABAR-expressing microglia *only* interact with inhibitory synapses? These cells also express 5-HT receptors as well as purinergic receptors that allow detection of highly active synapses. How do these signaling systems intersect with GABAR expression to shape specificity of microglial-synapse interactions?

## Non-phagocytic interactions between microglia and synapses

Microglia can also shape neuronal connectivity through non-phagocytic mechanisms (Weinhard et al., 2018). Microglial contact with dendrites promotes postsynaptic calcium elevations that increase likelihood of dendritic spine formation (Miyamoto et al., 2016). Conversely, microglial contact with dendritic spines can also increase likelihood of spine retraction, possibly *via* modification of local extracellular matrix and reductions in synapse stability (Tremblay et al., 2010; Nguyen et al., 2020). Microglial secretion of BDNF and TNFα can shape dendritic spine dynamics (Parkhurst et al., 2013) and AMPAR trafficking at synapses, respectively (Heir and Stellwagen, 2020). Finally, microglia can partially wrap around synapses and shape their function without progressing to full synapse engulfment, an interaction referred to as trogocytosis (Weinhard et al., 2018). Many of these non-phagocytic mechanisms for microglial interaction with synapses have been examined in a limited number of contexts and their involvement in maturation and refinement of many circuits, including mPFC circuits, remains unexplored.

Recent studies have identified yet more signaling pathways by which microglia can influence synapse structure and function. In the retinogeniculate system, there is a sensitive window of development (P20–P27) during which activity increases dendritic spine density in the dLGN (Cheadle et al., 2020). During this period, activity also increases microglial expression of the cytokine TWEAK and neuronal expression of its receptor Fn14. While Fn14 promoted spine formation, microglial TWEAK inhibited Fn14 function, acting to decrease spine density in dLGN neurons. Hence, in this system, microglia may exert precise control over synapse number, sensing neuronal activity and limiting synapse formation to keep neuronal excitability within an optimal range (Figure 1D). As Fn14-TWEAK signaling did not regulate engulfment of axon terminals by microglia and the complement system did not regulate spine density, separable microglial mechanisms may exist for regulation of pre- and postsynaptic synaptic compartments. New work shows that Fn14 is expressed in the deep layers of the developing frontal cortex (Ferro et al., 2021), suggesting microglia may use TWEAK-Fn14 to regulate activity-dependent circuit refinement there.

## Extrapolating lessons from sensory cortices to the mPFC

**FIGURE 2:**
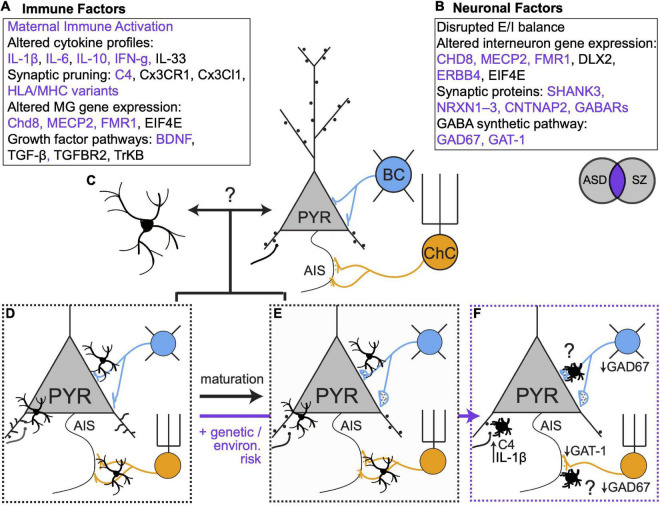
Genetic, environmental and microglial influences on synaptic connectivity in development and disease. **(A,B)** Immune and neuronal factors (Gauthier et al., 2010; Marín, 2012; Nieto et al., 2013; Volk and Lewis, 2013b; El-Ansary and Al-Ayadhi, 2014; McAllister, 2014; English et al., 2015; Estes and McAllister, 2015; Sinclair et al., 2016; Ishizuka et al., 2017; Gao et al., 2018; Jiang et al., 2018; Huang et al., 2019; Trossbach et al., 2019; Wiebe et al., 2019; Barbosa et al., 2020; Chen et al., 2020; Boukouaci et al., 2021) known to be altered in ASD and SZ. Purple text represents elements that are affected in both disorders. **(C)** Open question: to what extent do bidirectional microglia-neuron interactions regulate synaptic connectivity? **(D,E)**. Microglia regulate excitatory and inhibitory connectivity during healthy development. **(F)** In the context of genetic and environmental risk factors, microglia alter their phenotypes and contribute to altered synaptic connectivity. Many open questions remain, especially regarding the role of microglia in mediating maladaptive changes in inhibitory synaptic connectivity.

In the past decade, numerous studies have corroborated the idea that microglia sculpt developing neuronal circuits (Paolicelli et al., 2011; Schafer et al., 2012; Stevens and Schafer, 2018). Aligning the emerging work on microglial functions in sensory circuits with studies of mPFC synaptic development can help us develop testable predictions about how microglia regulate maturing mPFC circuits in health and disease (Figures 1E, 2). For example, in somatosensory cortex, classes of inhibitory cells and their synapses mature along different timelines. The number of chandelier cell synapses on the AIS peaks around P18 (Gallo et al., 2022), while the density of axosomatic basket cell synapses continues to rise until at least P30 (Favuzzi et al., 2021). Microglia shape this inhibitory landscape by promoting formation of axo-axonic chandelier synapses (Gallo et al., 2022) and pruning axosomatic synapses from PV + basket cells (Favuzzi et al., 2021), showing distinct interactions with individual synaptic compartments that are likely guided by the developmental status of the inhibitory neurons and their PyN targets. In mPFC, a similar time course of chandelier cell followed by basket cell maturation is observed (Miyamae et al., 2017). Hence, we may predict that mPFC microglia contact neuronal compartments to align their regulatory functions (synaptogenesis vs. pruning) to the developmental status of nearby synapses. However, depleting mPFC microglia in adolescence (P42–P52) produced the most profound changes in adult inhibitory synapses (Schalbetter et al., 2022), suggesting that microglial regulation of mPFC inhibitory synapses may be shifted to later time points than those observed in somatosensory cortex.

New work indicates that microglia use different signaling pathways to regulate dendritic spines depending on the stage of development (Cheadle et al., 2020; Yilmaz et al., 2021). In both sensory systems and mPFC, a period of synaptogenesis is followed by experience-dependent refinement, albeit along different timelines. After a wave of synaptogenesis in the dLGN (P0–5) (Schafer et al., 2012; Yilmaz et al., 2021), microglia prune presynaptic material and thin dendritic spines using complement-dependent phagocytosis (Yilmaz et al., 2021). Later, during experience-dependent refinement (P20-P27), microglia-secreted TWEAK reduces the density of bulbous spines using non-phagocytic mechanisms. In mPFC, microglial depletion before adolescence (beginning P28) reduces the density of long thin (presumably immature) spines while depletion during adolescence (beginning P42) reduced the density of mushroom (presumably mature) spines. These observations suggest that mPFC microglia may utilize distinct mechanisms to refine spines depending on the maturity level of individual synapses. Additional studies will be needed to clarify what defines whether and when microglia engage phagocytic or non-phagocytic mechanisms to regulate neuronal connectivity. Complement cascades play a role in adolescent mPFC synapse refinement (Yilmaz et al., 2021), but potential roles for Fn14-TWEAK and other non-phagocytic microglial-synapse interactions in mPFC remain to be determined.

## Roles of microglia in disease onset and progression

Microglia play a clear role in establishing and maintaining the exquisite balance of excitatory and inhibitory synapses that is required for proper circuit function. In ASD and SZ, dysfunctional social and cognitive behaviors have been linked to alterations in excitatory and inhibitory synaptic connectivity in mPFC (Volk and Lewis, 2013; Estes and McAllister, 2015; Schubert et al., 2015; Sohal and Rubenstein, 2019). Neuroimaging studies show reduced cortical gray matter and reduced spine density in individuals with SZ, consistent with the widely accepted hypothesis that excessive spine pruning in adolescence and early adulthood contributes to symptom onset (Glantz and Lewis, 2000). Convergent evidence also indicates that some features of SZ arise from deficits in cortical PV + interneurons (Lewis et al., 2005; Sohal et al., 2009; de Jonge et al., 2017). In the PFC of individuals with SZ, PV + interneurons show reduced expression of the GABA synthetic enzyme GAD67 and fewer PV + chandelier cells form cartridge synapses on the AIS (Rocco et al., 2017) of PyNs. Moreover, individuals with SZ show altered gamma oscillations –which depend critically on PV + cell activity (Sohal, 2012; Mathalon and Sohal, 2015)– during a working memory task (Barr et al., 2010). Reductions in mPFC spine density have also been described in mouse models of ASD (Bourgeron, 2015; Guo et al., 2019), and several ASD risk genes regulate inhibitory neuron function and inhibitory synapses. However, in contrast to the specific deficits in PV + neuronal function observed in SZ, inhibition may be affected more broadly in ASD (Chao et al., 2010; Marín, 2012). As an example, deleting the risk gene *MECP2* from all GABAergic interneurons recapitulated most behavioral features of Rett’s syndrome, a form of ASD, while deletion in specific regions or other broad cell types only recapitulated some features (Marín, 2012).

While a large body of work has identified perturbations in neuronal genes and proteins that affect synaptic connectivity (Figures 2A,B; Gauthier et al., 2010; Marín, 2012; Nieto et al., 2013; Volk and Lewis, 2013; El-Ansary and Al-Ayadhi, 2014; McAllister, 2014; English et al., 2015; Estes and McAllister, 2015; Sinclair et al., 2016; Ishizuka et al., 2017; Gao et al., 2018; Jiang et al., 2018; Huang et al., 2019; Trossbach et al., 2019; Wiebe et al., 2019; Barbosa et al., 2020; Chen et al., 2020; Boukouaci et al., 2021), accumulating evidence indicates that microglia are also key loci of dysfunction in neurodevelopmental disorders (Koyama and Ikegaya, 2015; Jin et al., 2017; Comer et al., 2020; Petrelli et al., 2020; Xu et al., 2020). In ASD and SZ, mPFC microglia have amoeboid morphology and increased cytokine production that is characteristic of an activated state (Fillman et al., 2013; Momtazmanesh et al., 2019). *C4* was recently identified as a SZ risk gene (Schizophrenia Working Group of the Psychiatric Genomics Consortium, 2014) and mutations in *C4B* have been linked to ASD (Estes and McAllister, 2015), suggesting microglia-mediated synaptic refinement is affected. Transcriptomic analyses of the brains of ASD patients revealed an enrichment of microglial modules (Gandal et al., 2018). Moreover, the studies we reviewed here highlight new mechanisms by which microglia can shape excitatory and inhibitory synaptic equilibrium and how changes in microglial gene expression can tip the balance of synaptic connectivity in pathological directions. Future studies that build on this work can determine how microglial functions are affected in diseases like SZ and ASD and will provide important insights into their normal developmental functions in mPFC.

In both SZ and ASD, it is thought that environmental challenges—such as infection or stress—occurring on a background of genetic risk can promote disease presentation (Estes and McAllister, 2015; Comer et al., 2020). However, the median age of onset is younger for ASD compared to SZ (Solmi et al., 2022), suggesting that in ASD, disease risk is intertwined with earlier-occurring developmental processes. This may explain why changes in cortical inhibition are more widespread in ASD whereas, in SZ, changes are more specific to mPFC PV + interneurons (Marín, 2012), which mature during adolescence (Du et al., 2018; Klune et al., 2021). Microglia are sensitive to environmental risk factors for SZ and ASD and early inflammatory events that increase risk for ASD and SZ can alter the developmental trajectory of microglia (Estes and McAllister, 2015; Parikshak et al., 2016). Moreover, the studies we discussed above demonstrate how microglia play age- and region-specific roles in spine (Cheadle et al., 2020; Yilmaz et al., 2021; Schalbetter et al., 2022) and PV-PyN (Favuzzi et al., 2021; Gallo et al., 2022) synapse maturation. As a result, insults that occur at different times likely affect distinct microglial-neuron interactions. In this way, microglia may determine, at least in part, when and where neuronal dysfunction arises in ASD and SZ, suggesting they are promising therapeutic targets.

## Looking ahead: Future research and unanswered questions

In healthy development, microglia and neurons undergo programmed developmental changes in their transcriptional profiles (Matcovitch-Natan et al., 2016; Hammond et al., 2019) and microglia exhibit region- and age-dependent differences in physical attributes and transcriptional status (De Biase et al., 2017; Hope et al., 2020). This underscores an urgent need to better understand the specific attributes of mPFC microglia across development—in healthy and diseased brains. The new studies we reviewed here suggest that microglia may tailor their functions to match the developmental status of local synapses and that microglial functions define sensitive windows of mPFC development. In the context of ASD vs. SZ risk, this implies that microglia may react to neuronal changes in synapse status and activity. Alternatively, transcriptional changes in microglia may be key drivers of neuronal dysfunction. Future studies that distinguish between these possibilities are needed to inform new therapeutic avenues (Figures 2C–F).

Most currently available tools for manipulation of microglia impact microglia throughout the CNS and caution is warranted in interpreting the impact of these manipulations on specific circuits. However, performing spatially restricted manipulations of microglia is challenging due to their resistance to infection with adeno-associated viruses (AAVs)—non-toxic viruses that are commonly used to manipulate gene expression in the brain. To advance the field, new approaches are needed for spatially restricted manipulation of these cells. In the meantime, as we learn more about the molecular mechanisms guiding neuron-glia interactions in mPFC, we can use neuronally-targeted AAVs to manipulate their interactions in a spatially and temporally restricted manner. With such tools in hand, we can determine when and how microglia regulate specific classes of mPFC synapses. Future studies can investigate to what extent microglia play simultaneous roles in pruning and synaptogenesis within mPFC—potentially at different classes of synapses—and whether microglia may toggle between states that promote synapse formation vs. pruning.

When investigating the roles of microglia in mPFC and sensory circuit development, it will also be important to consider potential contributions from astrocytes. Astrocytes shape circuit development in similar ways to microglia. Astrocyte-secreted proteins regulate synaptogenesis (Christopherson et al., 2005; Kucukdereli et al., 2011) and promote the clustering of glutamate AMPA receptors during synapse maturation (Allen et al., 2012). Moreover, astrocytes can phagocytically prune both excitatory and inhibitory synapses (Chung et al., 2013; Tasdemir-Yilmaz and Freeman, 2014). How do astrocytes and microglia coordinate their respective roles in circuit formation and refinement? Do they work in parallel or sequentially? Can each cell type compensate for the other in case of insults that damage one or both cell populations?

In addition to directly modulating synapse development, astrocytes signal to microglia to alter microglial function. For example, astrocyte-derived IL-33 signals to microglia to promote microglial synapse engulfment and neural circuit maturation in the thalamus and spinal cord (Vainchtein et al., 2018). Astrocytes can also release complement protein C3 in models of Alzheimer’s disease (Lian et al., 2015, 2016) raising questions about whether astrocytes secrete complement components in normal or pathological development with downstream consequences for microglial engulfment of synapses. Conversely, microglia can signal to astrocytes, with effects on synaptic function (Pascual et al., 2012) and astrocyte capacity to induce synaptogenesis (Liddelow et al., 2017). Based on these findings, microglia and astrocytes are likely to continuously regulate each other’s phenotype. A more comprehensive mapping of astrocyte-microglial signaling will be crucial to fully define how these glial cells shape neurodevelopment. Astrocyte-microglial communication also raises a cautionary note when interpreting microglial depletion experiments as providing evidence that microglia directly regulate synapses. Indeed, microglial depletion and repopulation can alter astrocyte gene expression (Coleman et al., 2020). Perturbation of astrocyte function has been implicated in ASD and SZ (Trépanier et al., 2016; Tarasov et al., 2020; Zhang et al., 2020; Notter, 2021; Wang et al., 2021; Allen et al., 2022) and astrocyte dysfunction in PFC has been implicated in inducing anxiety-like behaviors (Li et al., 2021) and cognitive impairment (Brockett et al., 2018; Petrelli et al., 2020), making the PFC of particular interest for studying astrocyte-microglial interactions.

## Concluding remarks

Studies from the past decade corroborate the notion that microglia and other immune system factors sculpt developing neuronal circuits (Paolicelli et al., 2011; Schafer et al., 2012; Stevens and Schafer, 2018). The work highlighted in this review demonstrates that microglia influence excitatory and inhibitory synapse maturation through a variety of complement-dependent, phagocytic and non-phagocytic signaling pathways. Further, microglial interactions with developing neural circuits seem to be tailored to specific classes of synapses, as well as the developmental stages of local neurons. Compared to our knowledge of their role in sensory system refinement, our understanding of how and when microglia regulate mPFC development remains limited. It is critical to address these knowledge gaps as mounting evidence links glial and prefrontal dysfunction to the same neuropsychiatric disorders, suggesting they might be causally related.

## Author contributions

All authors listed have made a substantial, direct, and intellectual contribution to the work, and approved it for publication.
